# Actinic Lichen Planus of the Forehead Mimicking Lentigo Maligna: A Case Report and Literature Review

**DOI:** 10.7759/cureus.89096

**Published:** 2025-07-30

**Authors:** Waseem Alhawsawi, Khalid A AlHawsawi, Abdulmohsin K Algethami, Wafi Al Hawsawi, Shaimaa H Banaji, Alhusain M Alshareef, Arwa Alharbi, Emtinan Alharbi

**Affiliations:** 1 Dermatology, King Fahad Armed Forces Hospital, Jeddah, SAU; 2 Dermatology, King Abdulaziz Hospital, Makkah, SAU; 3 Dermatology, King Abdulaziz Medical City, Jeddah, SAU; 4 Medicine, King Saud Bin Abdulaziz University for Health Sciences, Jeddah, SAU; 5 Dermatology, King Abdulaziz University Hospital, Jeddah, SAU

**Keywords:** actinic lichen planus, dermoscopy, lentigo maligna mimicry, lichen planus variants, pigmented facial lesions

## Abstract

We describe a 52-year-old Saudi male who presented with a six-month history of an enlarging pigmented macule over the left forehead. Clinical examination revealed a 1.4 × 2 cm asymptomatic black-grey patch with irregular borders and variable pigmentation. Dermoscopy demonstrated an irregular brownish network with multifocal areas of hyperpigmentation. The differential diagnosis included lentigo maligna, junctional nevus, and actinic lichen planus. A 4-mm punch biopsy showed hyperkeratosis, mild spongiosis, melanin incontinence, and perivascular lymphocytic infiltrates, findings highly suggestive of actinic lichen planus. No evidence of malignancy was observed. This case underscores the importance of including actinic lichen planus in the differential diagnosis of pigmented facial lesions in sun-exposed areas, particularly in patients with phototype III-V skin. Histopathology remains essential for diagnostic confirmation.

## Introduction

Lichen planus is a chronic inflammatory dermatosis with diverse clinical variants, including hypertrophic, atrophic, linear, and actinic subtypes. Actinic lichen planus is characterized by lesions confined to sun-exposed skin and was first described by Pock in 1961 [1]. Although most cases present as annular plaques with central clearing, pigmented macules resembling lentigo maligna have been reported [2]. This clinicopathologic overlap poses significant diagnostic challenges and may lead to unnecessary aggressive interventions if malignancy is suspected without biopsy confirmation. Here, we describe a case of actinic lichen planus of the forehead clinically mimicking lentigo maligna. Actinic lichen planus predominantly affects individuals of Middle Eastern, African, and South Asian descent and is typically diagnosed during middle age [3]. Ultraviolet radiation is believed to trigger a lichenoid reaction in predisposed individuals. Lesions are classically annular or plaque-like, hypopigmented or hyperpigmented, and localized to the face and dorsal hands [4]. Dermoscopy may reveal non-specific features such as pseudo-network pigmentation, making clinical differentiation from lentigo maligna or pigmented actinic keratosis unreliable [5]. Histopathology remains the gold standard, demonstrating lichenoid interface dermatitis with basal layer degeneration, hyperkeratosis, wedge-shaped hypergranulosis, and melanophages [6].

## Case presentation

**Figure 1:**
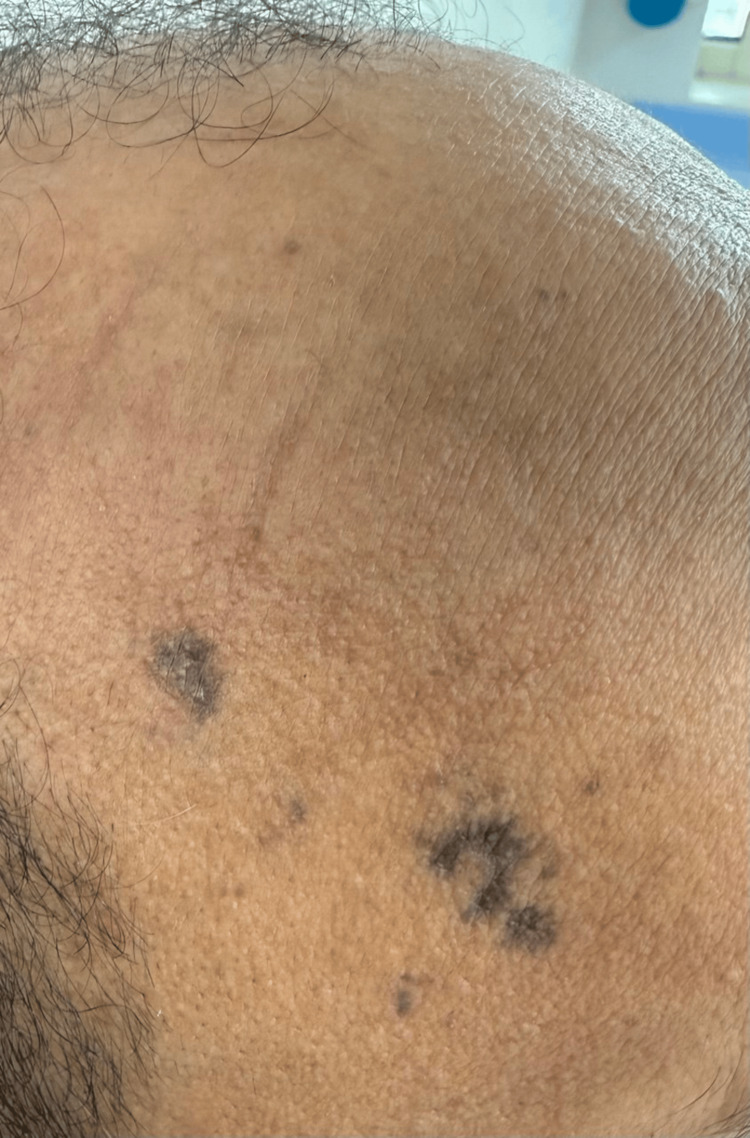
Morphology of the lesions showed darker reticulated patches on the right temple, and the lesions showed asymmetry and variation in shades of colors

**Figure 2:**
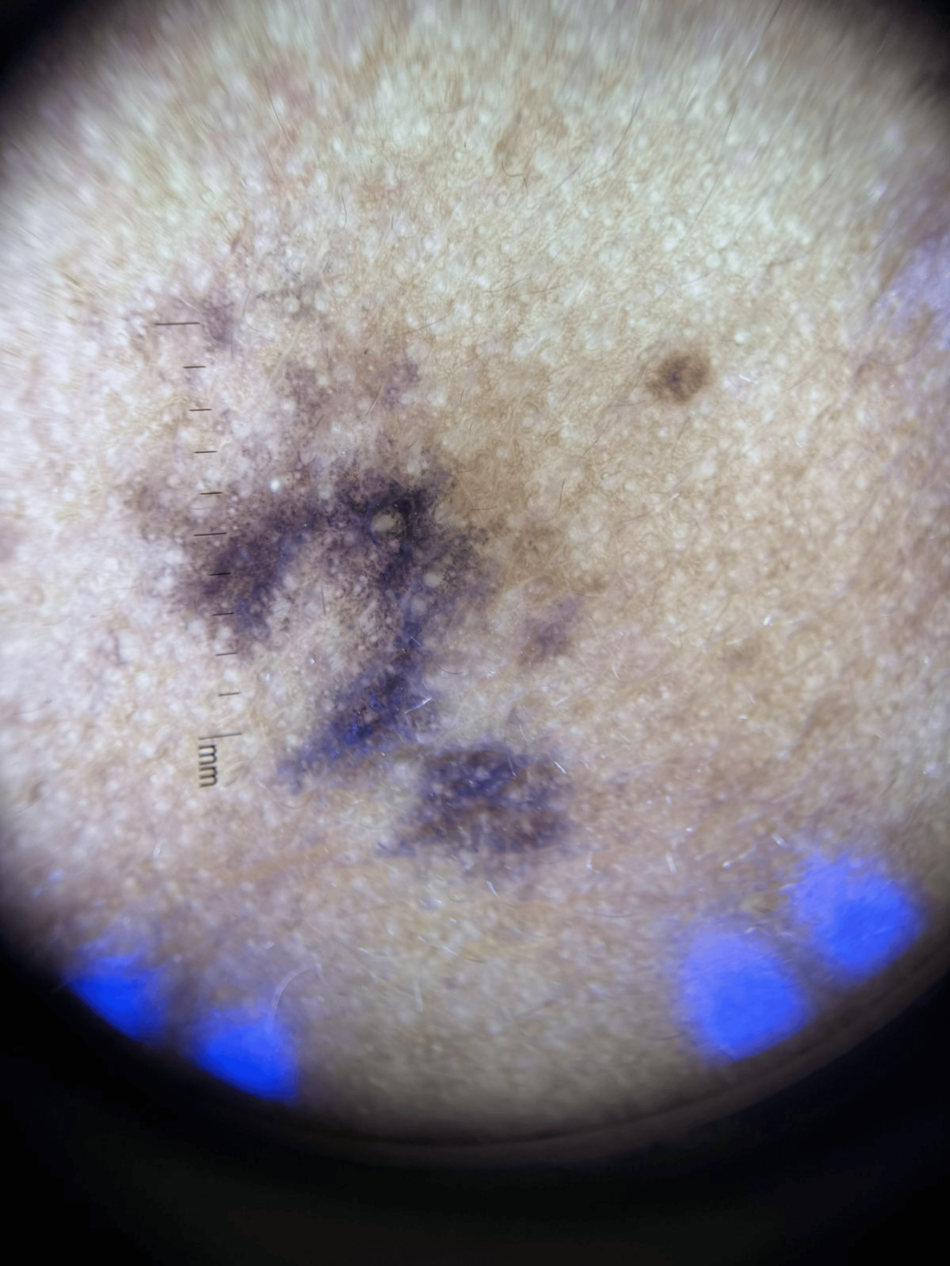
Dermoscopical image of the lesion showed a brownish globule forming a pseudo-network with agminated smaller circular whitish structures

A 52-year-old Saudi male presented to our dermatology clinic with a six-month history of a progressively enlarging pigmented lesion over the left paramedian forehead. The lesion was asymptomatic, with no associated bleeding, ulceration, or pruritus. His medical history was notable for type 2 diabetes mellitus managed with insulin therapy. The patient reported chronic occupational sun exposure, working outdoors for extended hours without consistent photoprotection. On clinical examination, a 1.4 × 2 cm irregular black-grey macule and patch was noted over the left forehead. The lesion demonstrated asymmetrical pigmentation with an ill-defined border and variegated shades of brown and grey (Figure 1). Dermoscopic evaluation revealed an irregularly reticulated brownish network interspersed with multifocal darker areas, raising suspicion for lentigo maligna or melanoma in situ (Figure 2). Given the atypical appearance, the differential diagnosis included lentigo maligna, actinic lichen planus, junctional nevus, and early melanoma in situ. To establish a definitive diagnosis, a 4-mm punch biopsy was performed from the most pigmented area. Histopathologic examination revealed hyperkeratosis and mild spongiosis within the epidermis, basal vacuolar alteration, and prominent melanin incontinence in the superficial dermis, accompanied by a superficial perivascular lymphocytic infiltrate. No atypical melanocytic proliferation or features of malignancy were observed. These findings were consistent with actinic lichen planus in the appropriate clinical context. The patient was counseled regarding rigorous photoprotection measures and initiated on high-potency topical corticosteroid therapy, with plans for close follow-up to monitor resolution and exclude any evolving malignant transformation.

## Discussion

The diagnosis of actinic lichen planus in this case highlights the remarkable clinical variability of lichenoid dermatoses. It underscores the potential for significant diagnostic confusion with premalignant and malignant pigmented lesions, particularly lentigo maligna. Actinic lichen planus is an uncommon photodistributed variant of lichen planus first described by Pock in 1961, typically affecting individuals in tropical and subtropical regions with prolonged sun exposure [1]. The precise pathogenesis remains incompletely elucidated, but ultraviolet radiation is believed to act as a trigger for a lichenoid immune reaction in genetically predisposed individuals [2]. Clinically, actinic lichen planus may present as annular plaques, hyperpigmented patches, or even discrete papules confined to sun-exposed areas such as the face, dorsal forearms, and hands [3]. In patients with darker skin phototypes, the pigmentation often predominates over erythema, which can obscure the classical violaceous hue and further confound clinical assessment [4]. In our case, the lesion’s irregular borders and variegated pigmentation simulated the appearance of lentigo maligna, leading to an initial clinical suspicion of melanoma in situ. Dermoscopy is an indispensable adjunctive tool in evaluating pigmented facial lesions but has limitations in differentiating lichenoid inflammatory disorders from melanocytic neoplasia [5]. The irregular pseudonetwork, asymmetrical pigmentation, and multifocal darker areas observed in our patient closely resembled lentigo maligna. This underscores that dermoscopy alone, while improving diagnostic confidence, cannot reliably exclude malignancy in atypical presentations, especially in chronically sun-exposed areas [6].

Histopathology remains the diagnostic cornerstone. The key histological features of actinic lichen planus include compact hyperkeratosis, mild spongiosis, vacuolar degeneration of the basal layer, melanin incontinence, and a band-like lymphocytic infiltrate in the superficial dermis [7]. These findings help distinguish it from lentigo maligna, which is characterized by proliferation of atypical melanocytes along the dermoepidermal junction and extension down adnexal structures [8]. Differentiating actinic lichen planus from other photodistributed dermatoses, such as discoid lupus erythematosus, lichenoid actinic keratosis, and pigmented actinic keratosis, is often challenging and requires clinicopathologic correlation [9]. Discoid lupus typically displays interface dermatitis with basement membrane thickening, follicular plugging, and prominent dermal mucin, features absent in our patient. Pigmented actinic keratosis and lentigo maligna may have overlapping dermoscopic and clinical features but lack the lichenoid infiltrate seen in lichen planus variants. Management of actinic lichen planus primarily involves rigorous photoprotection and topical corticosteroids. Recalcitrant cases may benefit from systemic immunosuppressive agents such as hydroxychloroquine or azathioprine [10]. In our patient, early histopathologic confirmation prevented unnecessary surgical intervention and facilitated prompt initiation of appropriate therapy. This case reinforces the critical importance of maintaining a broad differential diagnosis when evaluating pigmented lesions in sun-exposed skin, particularly in regions with high ultraviolet indices and among individuals with darker skin phototypes. Accurate diagnosis not only prevents overtreatment but also alleviates patient anxiety stemming from concern about malignancy. Additionally, this report highlights the need for clinician awareness of uncommon variants of lichen planus to improve diagnostic precision and optimize patient outcomes.

## Conclusions

Actinic lichen planus should be recognized as a distinct photodistributed variant of lichen planus that can closely resemble lentigo maligna on clinical and dermoscopic examination, especially in darker-skinned individuals and high UV-index regions. Histopathologic confirmation remains essential to avoid misdiagnosis and overtreatment, as it reliably distinguishes this benign inflammatory condition from malignant melanocytic proliferations. Management hinges on strict photoprotection and anti-inflammatory therapy, typically potent topical corticosteroids, though systemic agents like oral corticosteroids or acitretin may be warranted in persistent or extensive cases. With timely recognition and appropriate treatment, actinic lichen planus often resolves over months to years, preventing unnecessary surgical intervention and alleviating patient anxiety. This case underscores the need for histopathologic confirmation before definitive management of clinically suspicious pigmented macules, particularly in patients with darker phototypes and significant sun exposure.

## References

[1] Weston G, Payette M (2015). Update on lichen planus and its clinical variants. Int J Womens Dermatol.

[2] Venturini M, Manganoni AM, Zanca A (2018). Pigmented actinic lichen planus (PALP) mimicking lentigo maligna melanoma: usefulness of in vivo reflectance confocal microscopy in diagnosis and follow-up. JAAD Case Rep.

[3] Garg KS, Gratz BW, Lakeh B, Shahrour N, Tjahjono L (2025). Systematic review of lichen planus treatments for pediatric patients. JAAD Rev.

[4] Wagner G, Rose C, Sachse MM (2013). Clinical variants of lichen planus. J Dtsch Dermatol Ges.

[5] Stefanis AJ, Apalla Z, Papageorgiou C, Ioannides D, Nikolaidou C, Lallas A (2018). A tiny facial pigmented macule: overcoming the diagnostic challenge. Dermatol Pract Concept.

[6] Boch K, Langan EA, Kridin K, Zillikens D, Ludwig RJ, Bieber K (2021). Lichen Planus. Front Med (Lausanne).

[7] Ghosh A, Das A, Kumar D, Gharami RC (2014). Actinic lichen planus: a presentation deviant from the conventional. World J Dermatol.

[8] Stolz W, Schiffner R, Burgdorf WH (2002). Dermatoscopy for facial pigmented skin lesions. Clin Dermatol.

[9] Robles-Méndez JC, Rizo-Frías P, Herz-Ruelas ME, Pandya AG, Ocampo Candiani J (2018). Lichen planus pigmentosus and its variants: review and update. Int J Dermatol.

[10] Vincent JG, Chan MP (2015). Specificity of dermal mucin in the diagnosis of lupus erythematosus: comparison with other dermatitides and normal skin. J Cutan Pathol.

